# Texting-Based Reporting of Adverse Drug Reactions to Ensure Patient Safety: A Feasibility Study

**DOI:** 10.2196/publichealth.4605

**Published:** 2015-11-19

**Authors:** Godofreda Vergeire-Dalmacion, Nina T Castillo-Carandang, Noel R Juban, Maria Lourdes Amarillo, Maria Pamela Tagle, Emmanuel S Baja

**Affiliations:** ^1^ College of Medicine Department of Pharmacology and Toxicology University of the Philippines-Manila Manila Philippines; ^2^ College of Medicine Department of Clinical Epidemiology University of the Philippines-Manila Manila Philippines; ^3^ National Institutes of Health Institute of Clinical Epidemiology University of the Philippines-Manila Manila Philippines

**Keywords:** adverse drug reactions, pharmacovigilance, postmarketing, spontaneous reporting, texting

## Abstract

**Background:**

Paper-based adverse drug reaction (ADR) reporting has been in practice for more than 6 decades. Health professionals remain the primary source of reports, while the value of patients’ reporting is yet unclear. With the increasing popularity of using electronic gadgets in health, it is expected that the electronic transmission of reports will become the norm within a few years.

**Objective:**

The aims of this study are to investigate whether short messaging service or texting can provide an alternative or supplemental method for ADR reporting given the increasing role of mobile phones in health care monitoring; to determine the usefulness of texting in addition to paper-based reporting of ADRs by resident physicians; and to describe the barriers to ADR reporting and estimate the cost for setting up and maintaining a texting-computer reporting system.

**Methods:**

This was a pre-post cross-sectional study that measured the number of ADRs texted by 51 resident physicians for 12 months from the Department of Obstetrics and Gynecology and the Department of Adult Medicine of a tertiary government hospital in Manila, Philippines, with 1350-bed capacity. Reports were captured by a texting-computer reporting system. Prior to its implementation, key informant interview and focus group discussion were conducted. Baseline information and practice on the existing paper-based reporting system were culled from the records of the hospital’s Pharmacy and Therapeutics Committee. A postintervention survey questionnaire was administered at the end of 12 months.

**Results:**

Only 3 ADRs were texted by 51 resident physicians in 12 months (reporting rate 3/51 or 6%). By contrast, 240 ADRs from the paper-based reporting system from 848 resident physicians of the study hospital were collected and tabulated (reporting rate 240/848 or 28.3%). Texting ADRs was not efficient because of power interruption, competition with the existing paper-based reporting system, and unforeseen expiration of prepaid text loads/credits. The 3 ADRs texted were a report of vivid dreams and nightmares, a report of disturbing dreams and memory lapses, both of which were due to montelukast use, and a report of hepatitis from an isoniazid/rifampicin fixed-dose combination. Nineteen of 51 resident physicians (37%) registered in the reporting system responded to the postintervention survey. The most common reasons for not reporting ADRs were no adverse reaction identified 11/19 (58%) and restrictive reporting syntax 4/19 (21%). All doctors preferred a free form of reporting. The direct cost of the texting-based reporting system was calculated to be US $5581.40 and the indirect cost was US $9989.40. The total cost for texting-based ADR reporting system for 12 months was US $15,570.79.

**Conclusions:**

Reporting of ADRs via texting could be lower compared with an existing ADR paper-based system. Problems of Internet connectivity, reporting syntax, and expiration and reliability of text loads/credits should be addressed while implementing a text-based ADR reporting system in a developing country.

## Introduction

Toward the early 1970s, pharmacovigilance evolved as a critical field in drug development and regulation after the drug thalidomide showed serious adverse reactions in humans but not in animals. As an offshoot of this disastrous incident, in 1968, the World Health Organization (WHO) established the Program for International Drug Monitoring in Uppsala, Sweden [1]. The program created an adverse drug reaction (ADR) monitoring system based on spontaneous reporting by health care professionals. In the early 1980s, regulators, pharmaceutical manufacturers, and physicians realized that prolonging the approval of new drugs is as harmful as allowing marketing of drugs without postmarketing surveillance. Regulators also realized that rare ADRs, effects from drug-drug interactions, drug-disease interaction, and self-medication toxicities can only be elucidated in the real world of drug treatment rather than in clinical trials. This paved the way to the science of pharmacoepidemiology and the practice of pharmacovigilance.

To promote and encourage postmarketing surveillance, various strategies, regulatory policies, and even laws were created to facilitate the reporting of suspected ADRs. The major task for assuring drug safety was given to the pharmaceutical companies. However, this resulted in a conflict of interest and a restrained willingness to pass judgment on a drug’s culpability. A case in point is cerivastatin. After the release of cerivastatin on February 18, 1998, the dataset of ADRs grew, but important analyses of these data remained internal to Bayer Corporation [2]. Bayer modified the label of cerivastatin 5 times during the 3 years the drug was available to try to improve its safety before ultimately withdrawing it from the market. Reports of ADRs are often inadequately recorded or defined. This was proven by Loke and Derry in 2001 in their systematic review of the reporting and recording of ADRs in 185 randomized clinical trials. They found that 25 of the 185 trials (13.5%) did not mention anything about ADRs [3]. When ADRs such as clinical events or patient symptoms were mentioned in the reports, details on how they had been recorded were given in only 14 of 95 (15%) and 18 of 104 (17%) trials, respectively [3].

After 4 decades, postmarketing surveillance of newly marketed drugs has become a vital step in drug development, demanding the same attention and rigors as the other steps. It has also led to the science of pharmacovigilance, which is defined as “activities relating to the detection, assessment, understanding and prevention of adverse effects or any other drug-related problem.” Together with the WHO Collaborating Centre for International Drug Monitoring, Uppsala, WHO promotes pharmacovigilance at the country level. At the end of 2010, 134 countries have become part of the WHO pharmacovigilance program. Recently, pharmacovigilance has become synonymous with patient safety and care in relation to the use of medicines [1]. Although spontaneous ADR reporting is the mainstay of safety evaluation in the postapproval phase, a high level of unquantifiable underreporting by doctors remains an insurmountable problem of the system [4].

Another systematic review involving 37 studies across 12 countries was carried out in 2006 to numerically estimate the underreporting of ADRs using spontaneous reporting. The median underreporting rate was 94% (interquartile range 82-98%) [5]. There was no significant difference in the median underreporting rates calculated for general practice and hospital-based studies [5]. Despite more than 5 decades of spontaneous reporting, underreporting still remains a major drawback. Health professionals remain the primary source of reports, while the value of patients’ reporting is yet unclear. With the increasing popularity of using electronic gadgets in health, it is expected that the electronic transmission of reports will become the norm within a few years [6]. Ines in 1986 [7] published the earliest body of evidence on ADR reporting in the Philippines. This aforementioned study revealed that ADR reporting established in 1967 by the Philippine Medical Association has not been successful in gathering information on ADRs for the last 20 years of its existence. The author proposed in her study a change in the attitudes of patients and doctors toward ADRs and the adoption of newer strategy of reporting. Furthermore, a confidential and reliable mechanism of review and assessing the reports was recommended.

In the era of computers, cyberspace, and rapid connectivity, it is reasonable to explore the potential use of short messaging service, more commonly known as “texting,” in reporting ADRs. The use of mobile phones in health delivery services and care is not new. In 2008, mobile phone texting for pharmaceutical care in a hospital was implemented in China [8]. The system was called the “Mobile Pharmacy Service System.” The text messages sent by the system to patients consisted of the following: (1) reminders about medication from the day following discharge, (2) practical information about medicines, and (3) information about ADRs. Baron et al [9] published in 2013 a pilot study on the use of mobile phone-based tools for adverse event notifications after a vaccination program in Cambodia. A total of 184 patients from the study were texted for their clinical status 48 hours after their vaccinations. Fifty-two (28.3%) did not reply but 101 (54.9%) sent an immediate text response, and 31 (16.8%) sent a text reply after additional prompting. The study concluded that texting can also be a useful tool for notification by patients or health users in Cambodia, especially in an urban setting [9]. Local information on the texting activity of the Philippines in 2007 revealed that roughly 50 million are registered text message users and a staggering average of 195 texts were sent per user per month in the same year [10].

Our study aimed to test the usefulness of texting in conjunction with paper-based reporting of ADRs by resident physicians and to describe the barriers to ADR reporting; to determine the rate of texts reporting ADRs by resident physicians of 2 departments in a government tertiary care hospital; to determine the most commonly reported ADRs and the suspect drugs; and to estimate direct and indirect costs of texting for reporting ADRs.

## Methods

### Study Site and Design

This was a pre-post cross-sectional study with text-based ADR reporting system as the intervention. The study covered a period of 12 months from April 2011 to March 2012 excluding 3 months of preparatory work on the computer-texting system. The University of the Philippines-Philippine General Hospital (UP-PGH), a major government tertiary care hospital with 1350-bed capacity in the city of Manila, was chosen as the study site. The selection of the UP-PGH was based on the following criteria: presence of an active Pharmacy and Therapeutics Committee, high prescriptions based on annual patient admissions, a roster of health providers that remains relatively constant every year, and a higher probability of tracking down any patient reported to have an ADR. Two purposively selected study sites in the hospital, namely the Department of Adult Medicine and the Department of Obstetrics and Gynecology, were included in the study. The choice of these 2 departments was based on the expected high rate of prescribing of medications. Information materials and registration forms of the texting-based ADR reporting were disseminated 2 weeks prior to the launch of the project. Fifty-one resident physicians from the Department of Adult Medicine and the Department of Obstetrics and Gynecology signed the informed consent and were subsequently registered to the texting-based reporting system. Furthermore, information about the resident physician was entered in their registration directory, including their mobile phone numbers, age, gender, department, and year of residency. Key informant interview, review of hospital records, and focus group discussion (FGD) were conducted prior to the implementation of the texting-based reporting. After 12 months, a 2-paged survey questionnaire was administered to the 51 resident physician registered in the texting-based reporting to obtain their perception toward the new reporting strategy. In the same session, respondents were further probed about some of their replies to the questionnaire, which needed clarification. Moreover, during the entire study period, ADRs from the paper-based reporting system from all of the 848 resident physicians of the study hospital were collected and tabulated. In addition, direct and indirect costs were estimated using local currency and subsequently converted into US dollars.

### Creating the Internet-Based Reporting System With a Mobile Phone Interface

An information technology (IT) consultant was commissioned to create the texting-computer reporting system. It took 2 IT providers and almost 12 months of configuring the computer with the texting interface before the final form was ready for installation. The system utilized all the 3 local cellular phone companies that provide texting services in the country. All text messages received by any of the 3 mobile phone companies were automatically captured and sent to the database stored in the system. Anyone sending a report to the system was automatically acknowledged by a text message. The reporting system was given the acronym “DIMES,” or the “Drug Information and Monitoring Event for Safety.” A reporting syntax was required to be followed by the reporters for their texts to be accepted by the system. The database in the computer was also structured following the same order. The system was accessible 24 hours a day and 7 days a week and was configured to set an alert if there was a cluster of similar ADRs from one or more drugs or one drug repeatedly reported for several ADRs entering the system. The system administrator regularly reviewed the database for any signals or technical glitches. The research team used the Naranjo algorithm to produce a causality determination guideline for the study group [11]. The team also drafted a user’s manual to help the study group understand the technical features of the texting system and troubleshoot glitches. For the first 5 months of implementation, advisories on recent drug withdrawals and emerging profiles of new ADRs were sent to all texting-based registrants. These advisories were intended to serve as prompts indicating that the system was active and functioning.

## Results

### Result of the Key Informant Interview

Our key informant was a former director of the Philippine Food and Drug Administration (FDA). According to her, the Philippines was one of the earliest countries in Southeast Asia to become a member of the WHO Collaborating Centre for International Drug Monitoring in February 1995. The administration regularly sends ADR reports generated from its paper-based reporting system to Uppsala, Sweden. Initially, the members of the National Adverse Drug Reaction Advisory Committee assessed the reports for possible causality using global introspection. Our key informant directly supervised this committee’s operation for many years before her retirement. According to the key informant, the committee disbanded in 2006, after which almost no ADR reports were recorded. In 2007, the Philippine FDA launched a WHO-supported pharmacovigilance strategy, “Bantay Gamot,” or “Drug Watch.” Drug Watch is a paper-based consumer-reporting scheme, which continues to effectively receive reports on the substandard quality of drugs. In 2010, the Philippine FDA collaborated with Department of Health Information Management System to establish an online ADR reporting system. However, many health professionals and pharmacovigilance officers from drug companies reported difficulty with opening the site, a major drawback to its effectiveness. In 2011, the Philippine FDA received 2032 ADR reports, of which 691 were sent to Uppsala, Sweden. The UP-PGH’s Pharmacy and Drug Committee was one of the regular contributors to the Philippines FDA.

### Result of the Focus Group Discussion

Ten resident physicians from the 2 study sites participated in the FGD. A summary of the findings from the discussion is shown in Textbox 1.

**Results of the focus group discussion describing the knowledge, attitude, and practice of resident physicians toward drug prescribing and adverse drug reactions.**

Knowledge of drug prescribing

- The most commonly prescribed drugs were antihistamines, steroids, chemotherapeutics, ferrous sulfates, contraceptives, metronidazole, insulin, and analogues.
- The usual number of drugs taken per patient is between 2 and 3.
- 75% of prescriptions are orally administered.

Knowledge of ADRs

- Overall, ADRs are very rare and are usually managed on an outpatient basis.
- Common types of reactions are anticipated side effects (9 of 10), and unforeseen events (1 of 10).
- ADRs from contrast media should be included as source of ADRs.
- The issue of therapeutic failure due to substandard generics should be included.

Physicians’ knowledge and attitude toward patients reporting ADRs

- Patients already report adverse reactions to their doctors using text messaging mostly about their follow-ups or consulting earlier than scheduled.
- Patients usually consult for drug-related allergies.
- Female patients (including female relatives of male patients) report ADRs more than males.
- Doctors believe that patients are not in a good position to report ADRs.
- Physicians tend to only ask about known side effects.

Attitude toward reporting of ADRs

- Possible barriers to reporting ADRs include loss of the plan/texting ability due to payment/credit expiration, no mobile phone, no transportation money, paperwork, no signal, rejection of texts because they are too long.
- Factors facilitating adverse drug response reporting are good rapport with the doctor, clear symptoms, giving the responsibility to female relatives, use of a specific telecommunications provider, and providing feedback to the doctor.
- Participation concerns regarding the texting-based system include availability of journal articles as support to alerts, assurance that the texting-based system will not replace the doctor or pit one doctor against another.

All resident physicians were receptive to using texting to report ADRs. They also suggested using texts to send clinical evidence from journals. Not surprisingly, all the participants had used texting to remind patients of their clinic visits and to reply to patients’ queries about dosing and drug administration. Most agreed that reporting is time consuming and all reporters expect to receive feedback on their reports.

### Results of Texting-Based and Paper-Based Reporting

**Figure 1:**
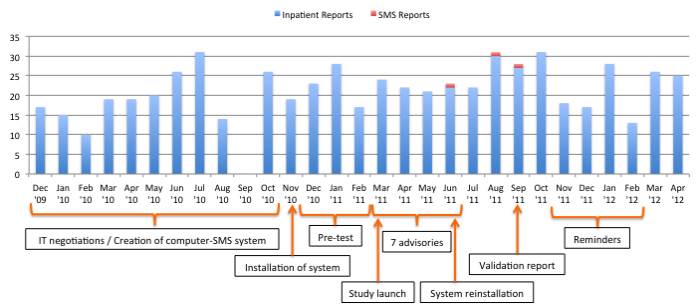
Timeline of the study before and immediately after the implementation of the texting-based reporting system and the adverse drug response reports received during this period. SMS: short messaging service.

Figure 1 shows the ADR results of both the texting-based and paper-based reporting systems in the hospital. A total of 277 ADR reports from the paper-based system of the UP-PGH were recorded the year before the implementation of the texting-based reporting system. No paper-based ADR report was available in September 2010. The reports came from the 848 resident physicians of all departments and sections of the hospital. The total number of resident physicians in the entire hospital is basically the same through the years due to the fixed number of positions in each department and section of the hospital. The reporting rate from the paper-based system prior to the implementation of the texting-based system was 32.7% (277/848).

During the implementation of the texting-based system, a total of 240 ADR reports were recorded from the paper-based system giving a reporting rate of 28.3% (240/848). However, the texting-based reporting system obtained only 3 ADR reports from the 51 resident physicians of the 2 preselected specialty departments of the study hospital, which translated to a reporting rate of only 6% (3/51). Most of the ADRs received by the hospital from the paper-based reports were liver dysfunctions and jaundice from antituberculosis drugs and reports of allergies. Conversely, ADR reports from texting-based system consisted of one report of vivid dreams and nightmares and one other report of disturbing dreams and memory lapses both from montelukast. There was also one report of hepatitis from an isoniazid/rifampicin fixed-dose combination.

### Limitations of the Texting-Computer Hardware of the Reporting System

Numerous technical glitches were encountered, mostly brought about by fluctuating mobile phone connectivity, frequent power interruptions, and insufficient phone loads/credits. Unfortunately, these episodes of disconnection were not observed during the 2-3 months of pretesting the system; they only became evident toward the middle of the study. There were no explanations for the absence of cellular connectivity on certain days. In addition, prepaid loads/credits of the phones were already used up even 2-3 weeks before their expiration dates. To decrease the likelihood of missed reports, 1 cellular company, which was not the service provider of any of the 51 registered resident physicians, was removed from the system. The removal was done to decrease the number of phones that needed to be reloaded. Expiration of text loads/credits could have been avoided had the system been enrolled in a postpaid plan. A postpaid plan entitles the subscriber to a 4-digit mobile phone number that is easily remembered. Unfortunately, a subscriber needs to have 20,000 Philippine pesos (PhP) worth of text messages or calls per month to qualify for a postpaid plan.

### Postintervention Activity

Table 1 summarized the results from the postinterventions survey among resident physicians who registered in the texting-based system. Only 37% (19/51) submitted the completed questionnaire. The major reason for not reporting ADR was the absence of an identifiable ADR (11/19; 58%) followed by the restrictive reporting syntax (4/19; 21%).

**Table 1:** Postintervention survey among resident physicians registered in the text reporting on their perception toward the adverse drug reaction texting-based reporting (N=19).

Survey questions		n (%)
**Resident physicians who received advisories**		
	Yes	12 (63)
	No	5 (26)
	No reply	2 (11)
**Reasons for not reporting via text**		
	No identified ADRs	14 (74)
	Reported through paper based	1 (5)
	No answer	4 (21)
**Reasons for not reporting ADR via any reporting method**		
	No identifiable ADRs	11 (58)
	Constraining text syntax	4 (21)
	Presence of paper-based	4 (21)
	Lack of time	1 (5)
	Lack of perceived need	1 (5)
	Fear of litigation	0 (0)

### Cost Estimates

The direct and indirect costs of the text-based reporting system were computed. For the direct cost, the cost of the computer, 3 mobile phones, and a one-time cost of the subscriber identification module (SIM) cards were included in the computation. The total direct cost was 240,000 PhP or US $5581.40 at an exchange rate of US $1 to 43.00 PhP. The indirect cost included the prepaid phone loads for 12 month at 19,544.00 PhP (US $454.51), the IT consultancy fee of 50,000.00 PhP (US $1162.79), and the honorarium of the system administrator of 360,000.00 PhP (US $8372.09) for 12 months. The total indirect cost was 429,544.00 PhP (US $9989.40). Adding the direct and indirect costs, the total estimated cost was 669,544.00 PhP or US $15,570.79 to establish and run the texting-based reporting for 1 year.

## Discussion

### Preliminary Findings

The Philippine FDA handles the Adverse Drug Reaction Spontaneous Reporting System in the Philippines. According to our key informant, underreporting of ADRs has perennially hampered the reporting system in the Philippines. Historically, the system has received only 1000-3000 reports per year. Yet, in 2003, drug sales from the largest drug retailer in the Philippines amounted to nearly 43 billion PhP [12]. ADR reporting to vary from 7% of all hospital admissions in the UK to 13% of all admissions in medical clinics in Sweden [13]. In New Zealand, 12.9% of all hospital admissions were due to adverse drug events [13,14]. To improve the detection of previously unknown serious ADRs, the US FDA introduced the MEDWATCH program in 1993 [13]. Approximately 1 year from its introduction, the number and quality of ADR reports to the FDA increased. However, this rise was attributed to increased reporting from pharmacists. Physician reports declined slightly during this period. Although the medical literature is rich on studies about ADRs, there is none on the use of texting as a strategy for reporting them. To our knowledge, our study is the first look at texting as an alternative method of reporting ADR. In addition, our study was conducted on the premise that reporting of ADRs can be improved with the use of a ubiquitous and popular communication technology such as texting. However, our study found no increase in the number of ADR reports using texting. Our study also revealed that texting was highly dependent on a reliable telecommunication services, which entails a relatively large amount of monthly postpaid plan of 20,000.00 PhP or US $435.00. In addition, efficient syntax reporting plays a critical role in reporting ADR in texting-based reporting system.

The reporting rate from the texting-based system (3/51; 6%) of our study was absolutely lower compared with the reporting rate from the paper-based system (240/848; 28.3%). Our finding should be regarded with caution because the calculation of the reporting rate for both the texting-based and paper-based systems used a different total number of resident physicians (51 vs 848). The texting-based system only received ADR reports from 2 departments of the study hospital, whereas the paper-based system covered all specialty departments and sections of the hospital. These 2 departments were purposively selected because of their higher use of medications compared with purely surgical departments. The reporting rates were used simply to describe the state of both reporting system. Nevertheless, more studies are needed to verify and validate the efficiency of a texting-based reporting system as an alternative system in ADR reporting.

Brewer and Colditz in 1999 [15] observed that spontaneous reporting systems could be effective in revealing unusual and rare events that occur with the use of medications. However, they showed that spontaneous reporting systems were not reliable for detecting ADRs occurring far from the time of intake or in a population not commonly exposed to the drug. Brewer and Colditz [15] recommended the use of other methods, such as clinical trial data, medical records, and computerized databases of medication users and nonusers to complement spontaneous reporting. Huang et al [16] reviewed in 2014 the postmarketing drug surveillance for adverse drug events worldwide. It showed 2 systems of surveillance in the UK using administrative claims or electronic medical records and most pharmacovigilance being conducted on behalf of a regulatory agency. To access existing data, either a common data model or a centralized model could be used. Aside from studying existing databases as data sources for detecting ADRs, methods for reporting ADR such as texting were explored by our study. However, results from our study showed that texting could be unreliable due to consumable phone loads/credits and an unpredictable power supply. Nonetheless, these obstacles may be less common in more economically developed countries.

Resident physicians were probed about their reply of “no identified ADR” as a reason for not reporting. About a third of the respondents did not find it necessary to report known and accepted adverse reactions to a suspect drug. The rest really admitted to not identifying any ADR during the 12-month study period. The observation that few ADR reports were due to “unrecognized adverse events” was likewise reported in a study by Hartigan-Go in 2002 [17]. Hartigan-Go [17] reported that sometimes the adverse event is misconstrued as part of the healing action. This suggests that physicians and other health professional prescribing medications should be regularly educated on the principles and rationale of pharmacovigilance and be reminded on how important it is to quantify even known ADRs to drugs in their local clinical setting. Contrary to our expectations, fear of litigation was never considered a deterrent to reporting.

However, there is still room for studying the use of texting for reporting ADR by other health professionals, such as nurses and clinical pharmacists, provided the observed obstacles and problems are resolved. More people reporting will help generate the 20,000.00 PhP required for the postpaid plan that will ensure uninterrupted Internet service. Lastly, texting might be best suited for timely dissemination of drug information bulletins, drug advisories, or as a tool for reminding patients on their follow-up visits. A study by Kew in 2010 [18] found that texting via mobile phone was an effective method for collecting weekly symptom reports during a clinical trial, reminding trial patients to attend face-to-face visits and completing more complex paper-based evaluation.

### Conclusions

In summary, the reporting rate of ADRs using texting-based ADR reporting system may be lower compared with the paper-based ADR reporting system. Unreliable telecommunication services, frequent electrical interruptions, reporting syntax, and expiring prepaid loads/credits should be addressed when setting up a texting-based ADR reporting system in a developing country.

## Abbreviations

ADR: adverse drug reaction
FDA: Food and Drug Administration
FGD: focus group discussion
IT: information technology
SIM: subscriber identification module
UP-PGH: University of the Philippines-Philippine General Hospital
WHO: World Health Organization
